# Non-linearity of the collagen triple helix in solution and implications for collagen function

**DOI:** 10.1042/BCJ20170217

**Published:** 2017-06-16

**Authors:** Kenneth T. Walker, Ruodan Nan, David W. Wright, Jayesh Gor, Anthony C. Bishop, George I. Makhatadze, Barbara Brodsky, Stephen J. Perkins

**Affiliations:** 1 Department of Structural and Molecular Biology, Darwin Building, University College London, Gower Street, London WC1E 6BT, U.K.; 2 Center for Biotechnology and Interdisciplinary Studies, Rensselaer Polytechnic Institute, 110 8th Street, Troy, NY 12180-3590, U.S.A.; 3 Department of Biomedical Engineering, Science and Technology Center, Tufts University, 4 Colby Street, Medford, MA 02155, U.S.A.

**Keywords:** atomistic modelling, collagen, molecular dynamics, small-angle scattering, ultracentrifugation

## Abstract

Collagen adopts a characteristic supercoiled triple helical conformation which requires a repeating (Xaa-Yaa-Gly)_n_ sequence. Despite the abundance of collagen, a combined experimental and atomistic modelling approach has not so far quantitated the degree of flexibility seen experimentally in the solution structures of collagen triple helices. To address this question, we report an experimental study on the flexibility of varying lengths of collagen triple helical peptides, composed of six, eight, ten and twelve repeats of the most stable Pro-Hyp-Gly (POG) units. In addition, one unblocked peptide, (POG)_10unblocked_, was compared with the blocked (POG)_10_ as a control for the significance of end effects. Complementary analytical ultracentrifugation and synchrotron small angle X-ray scattering data showed that the conformations of the longer triple helical peptides were not well explained by a linear structure derived from crystallography. To interpret these data, molecular dynamics simulations were used to generate 50 000 physically realistic collagen structures for each of the helices. These structures were fitted against their respective scattering data to reveal the best fitting structures from this large ensemble of possible helix structures. This curve fitting confirmed a small degree of non-linearity to exist in these best fit triple helices, with the degree of bending approximated as 4–17° from linearity. Our results open the way for further studies of other collagen triple helices with different sequences and stabilities in order to clarify the role of molecular rigidity and flexibility in collagen extracellular and immune function and disease.

## Introduction

Collagen is the most abundant protein in the human body, providing required structural and mechanical properties, as well as cell signalling, to tissues such as tendon, skin and cartilage. Collagen is also found as a domain in a range of host-defense proteins including C1q, collectins, and macrophage scavenger receptors. The characteristic molecular structure of collagen is the triple helix, a specialized protein structure motif. The triple helix is composed of three polyproline II-like polypeptide chains staggered by 1 residue with respect to each other [1–3]. The three chains are supercoiled about a common axis in a right handed manner, with backbone NH (Gly)…CO hydrogen bonds formed between the chains. The close packing of the three chains can accommodate only the smallest residue Gly in the center, generating the requirement for Gly as every third residue in the triplet repeats of the collagen amino acid sequence, (Xaa-Yaa-Gly)_n_. The residues in the Xaa and Yaa positions are largely exposed to solvent, and are frequently occupied by the imino acids Pro (in the Xaa position) or the post-translationally modified hydroxyproline (Hyp or O, in the Yaa position), which further stabilize the triple helix. The unique supercoiled triple helix conformation forms an elongated rod-like domain, which often associates to form fibrils or other supramolecular structures in many biologically important collagens and host defence proteins [4,5].

The rod-like nature of the collagen molecule and its dimensions (∼1.5 nm diameter and 300 nm long) were established in early physical chemical studies of collagen [6]. Diverse experimental approaches, including sedimentation, viscosity, viscoelastic measurements and light scattering studies, as well as direct microscopic visualization and computational approaches suggested that the collagen triple helix is not fully rigid [7–12]. A wide range of persistence lengths have been reported for collagen by different techniques, and their values range from those consistent with a random coil to those expected for an almost rigid structure. These studies were much enhanced by studies of synthetic peptides that adopted the collagen triple helical conformation because Gly was present as every third residue, the imino acid content was sufficiently high, and the peptide was of sufficient length [13]. The tripeptide Pro-Hyp-Gly (POG) is the most stabilizing sequence found in collagens and peptides based on this sequence have been extensively studied in terms of stability, structure and dynamics [13]. Thus high resolution X-ray crystallography studies of peptides with repeating sequence (Pro-Hyp-Gly)_n_ have defined the molecular details of a linear structure for the triple helix [14,15]. For example, the high resolution structure of (Pro-Hyp-Gly)_10_, also known as (POG)_10_, has a precise 7/2 superhelical symmetry, and details of the imino acid ring pucker and hydration have been determined [3,15]. NMR relaxation parameters and hydrogen-exchange studies for (Pro-Hyp-Gly)_10_ and a peptide with an imino acid poor collagenase cleavage region have been reported [16], but it is not clear how such highly localized dynamic features relate to overall triple helix flexibility.

Determining whether the rod-like triple helix is rigid or flexible by advanced molecular modelling will improve our understanding of structure-function relationships and disease-causing mutations in collagens and immune defense proteins [4,5]. Here, a recently developed atomistic modelling approach to monitor the bending for heparin, heparan sulfate and other linear polymers of increasing length was adapted to define molecular structures for well-characterized collagen triple helices [17]. Firstly, the degree of flexibility as a function of length for (POG)_n_ collagen triple helices in solution was monitored by a combination of analytical ultracentrifugation (AUC) and small angle X-ray scattering (SAXS). These methods provided experimental data for five model collagen helices, namely the blocked peptides (POG)_6_, (POG)_8_, (POG)_10_ and (POG)_12_, and the unblocked peptide (POG)_10_. Secondly, molecular dynamics (MD) methods were used to determine 50 000 physically-realistic atomistic models for each of these five triple helices. The filtering of these models against the experimental SAXS data identified a small family of best-fit structures in solution, to follow recent other similar studies [18,19]. Our combined experimental and atomistic modelling evidence demonstrated for the first time the existence of non-linearity in detailed molecular models of collagen triple helices as their lengths increased, and provided representative molecular structures for these (Supplementary Information). These results will be key for future studies on the flexibility within biologically active or mutated collagen sequences and clarify the role of molecular rigidity and bending in collagen function and disease.

## Materials and methods

### Production and preparation of collagen peptides

The peptide (Pro-Hyp-Gly)_10_ with both ends unblocked was obtained from Peptides International (Louisville, Kentucky). All other peptides were synthesized by the Tufts University Core Facility (Boston, MA), and were blocked at both ends (acetylated N-terminus; amidated C-terminus). All peptides were purified using a reverse-phase HPLC system on a C-18 column, confirming a purity of >95%. The identity and molecular mass of the peptides were confirmed by laser desorption mass spectrometry. Peptide concentrations were measured by absorbance at 214 nm using ɛ^214^ = 2200 cm^−1^ M^−1^ per peptide bond.

### Sedimentation velocity data for collagen helices

AUC data were obtained on two Beckman XL-I instruments equipped with An-60Ti analytical rotors and using two-sector cells with column heights of 12 mm at a rotor speed of 60 000 rpm. Sedimentation velocity experiments were performed at 20°C, at three concentrations between 1.6–2.6 mg/ml for (POG)_6_, 0.4–4.9 mg/ml for (POG)_8_, 1.39–3.97 mg/ml for (POG)_10_, 1.20–3.6 mg/ml for (POG)_12_ and 1.09–3.5 mg/ml for (POG)_10unblocked_ and in 137 mM PBS (137 mM NaCl, 2.7 mM KCl, 1.4 mM KH_2_PO_4_, 4.3 mM NaH_2_PO_4_, pH 7.4). Prior to experiments, the helices were formed by taking the peptides up in PBS and allowing the solution to equilibrate for at least two hours prior to measurement. Sedimentation was monitored using absorbance optics at 232 and 225 nm. The sedimentation coefficient distribution *c*(*s*) analyses were performed by fitting up to 45 absorbance scans directly to the Lamm equation using SEDFIT software version 14.6e [20,21]. In the *c*(*s*) analyses, the meniscus, the bottom of the cell, the baseline, and the average frictional ratio *f*/*f*_0_ were floated until the overall root mean deviation and the fits between the observed and calculated sedimentation boundaries were satisfactory. The *f*/*f*_0_ values used were 1.04 for (POG)_6_, 1.08 for (POG)_8_, 1.17 for (POG)_10_, 1.32 for (POG)_12_ and 1.04 for (POG)_10 unblocked_. The partial specific volume *ṽ* was calculated to be 0.735 ml/g for all (POG)_n_ peptides [20]. The buffer density was 1.00543 g/ml, measured using an Anton Paar DMA 5000 density meter.

### X-ray scattering data for collagen helices

SAXS data were acquired in two beam sessions on the BioSAXS robot at Instrument BM29 at the European Synchrotron Radiation Facility, Grenoble, France [23,24]. Data were recorded using a CMOS hybrid pixel Pilatus 1M detector with a resolution of 981 × 1043 pixels (pixel size of 172 µm × 172 µm). Both sessions were operated with a ring-energy of 6.0 GeV in 16-bunch mode. The sample-detector distance was set to 2.864 m, the X-ray wavelength was 0.09919 nm, and the diameter of the flow cell quartz capillary was 1.8 mm in both sessions. Potential radiation damage was averted by the continuous movement of the sample in the flow cell during beam exposure; the use of 10 time frames with an exposure time of 1 s per frame; and on-line checks for the absence of radiation damage. The scattering data were collected for (POG)_6_ at 0.25–2.64 mg/ml and for (POG)_8,_ (POG)_10,_ (POG)_12, and_ (POG)_10 unblocked_ at 0.25–1.00 mg/ml in 137 mM NaCl PBS buffer at 20°C. Data reduction was performed using ISPyB software [25].

In a given solute-solvent contrast, the radius of gyration *R_g_* corresponds to the mean square distance of scattering elements from their centre of gravity, and is a measure of structural elongation. Guinier analyses at low *Q* values (where *Q* = 4π sin θ/*λ*; 2*θ* is the scattering angle and *λ* is the wavelength) give the *R_g_* value and the forward scattering at zero angle *I*(0) from the expression [26]:

$$\ln\;I(Q)=\ln\;I(0)-\frac{R_{g}^{2}Q^{2}}{3}.$$

This expression is valid in a *Q.R_g_* range up to 1.3. GNOM software was used to calculate a real space estimation of the *R_g_* [27].

### Generation of linear collagen helices for comparisons against AUC and SAXS data

Linear models for the collagen helices were constructed from a linear crystal structure (PDB code 3B0S), which has a 7/2 helical conformation and a (GPO)_9_ repeat unit [15]. Linear models of (POG)_10_ and (POG)_12_ were created through duplication and superimposition of the crystal structure, followed by the removal of excess residues. (POG)_6_ and (POG)_8_ were created through the removal of excess residues from the (GPO)_9_ structure.

### MD simulations of collagen helices

Initial triple helices based on all-atom (POG)_n_ triplets, where *n* = 6, 8, 10 and 12, were generated using the THeBuScr version 1.07 software package [28]. The N- and C-termini were then subsequently blocked with acetyl and amide groups respectively. For the (POG)_10unblocked_ simulation, this step was omitted. The models were energy minimized using the steepest descent algorithm and then placed in a TIP3P-solvated, cubic box with an edge length 0.7 nm larger than the largest axis of the model. Following solvation, the systems were energy minimized. Then the systems were equilibrated by running short, subsequent 1 ns simulations at 50, 100, 150, 250 and 300 K, such that the final structure from the previous simulation was used as the starting structure for the next simulation. Following temperature equilibration, a production MD was run at 300 K utilizing a 2 fs time step for a simulation time of 50 ns for each of the collagen models. Extending the simulations to longer time scales (up to 200 ns) did not qualitatively change the results of the *R_g_* analyses. All molecular dynamics simulations were carried out using the GROMACS v4.5.5 software package with the AMBER99sb-ILDNP forcefield [29]. Other simulation details were the same as described by us previously [30].

### SAXS curve calculation using SCT

Scattering curves were calculated from the MD snapshot structures using the open source SCT software [31]. The atomic co-ordinates for each structure were coarse grained into sphere models, using a grid with a box size of 0.53 nm and a cut off of four atoms. The hydration shell bound at the protein surface contributes to the SAXS curves at a similar level to the protein, and this was modelled by the addition of hydration spheres corresponding to 0.3 g of water per gram of protein [22,32]. Scattering curves were calculated using the Debye equation adapted to spheres. The experimental and calculated scattering curves were compared through the calculation of *R* factors:

$$R\;\mathrm{factor}=\frac{\sum ∥\left||I_{\mathrm{Expt}}(Q)||-\eta ||I_{\mathrm{Theor}}(Q)|\right|∥}{\sum ||I_{\mathrm{Expt}}(Q)||}\times 100$$

Similar to crystallography, low *R* factors represent the better fit structures.

## Results

**Figure 1.**
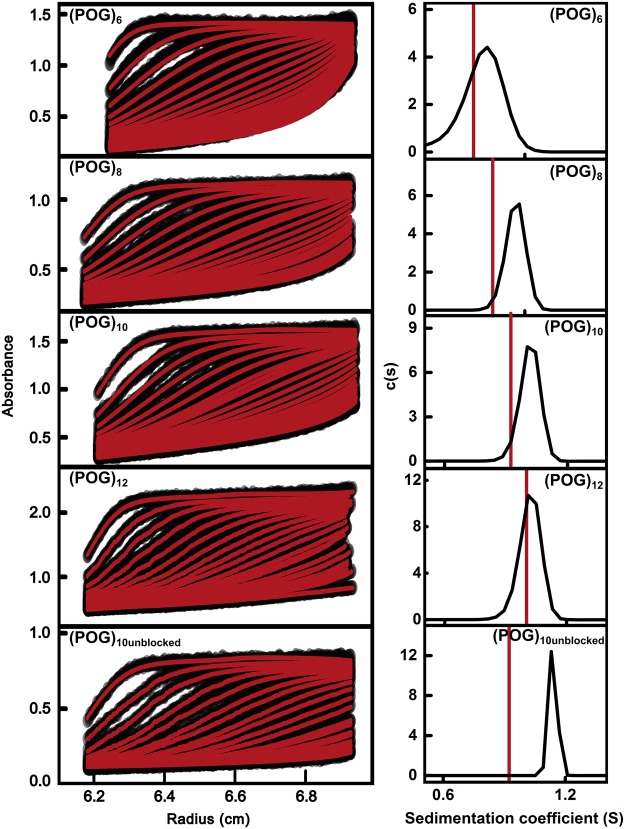
Sedimentation velocity analyses for the five collagen peptides. Sedimentation boundaries for five collagen peptides (POG)_6_, (POG)_8,_ (POG)_10_ and (POG)_12_ and (POG)_10-unblocked_ were fitted using SEDFIT. Absorbance data were collected at 232 nm at a rotor speed of 60 000 rpm with concentrations of 1.5–2.6 mg/ml. The left panels show the fitted sedimentation boundaries for (POG)_6_ - (POG)_12_. The right panels show the individual size-distribution analyses *c*(*s*). These analyses produced *s_20,w_* peaks at 0.81 S for (POG)_6_, 0.99 S for (POG)_8_, 1.06 S for (POG)_10_, 1.18 S for (POG)_10unblocked_ and 1.06 S for (POG)_12_. The red line in each panel denotes the theoretical *s_20,w_* value for the linear crystal-derived collagen model.

**Figure 2.**
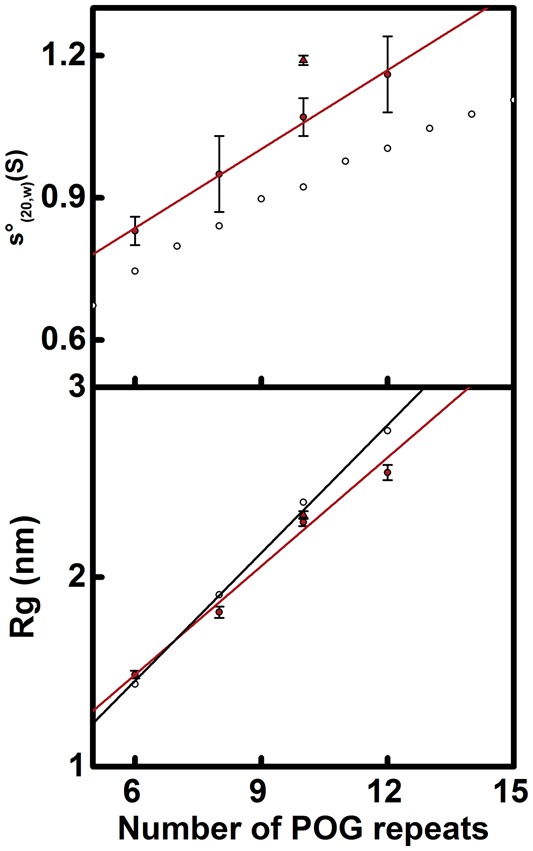
Comparison of experimental and crystallographic *s*_(*20,w*)_ and *R_g_* values. The top panel compares *s_20,w_* values against peptide length. The filled circles represent the experimental values for (POG)_6_, (POG)_8,_ (POG)_10_ and (POG)_12_. (POG)_10unblocked_ is represented by a filled triangle. The four unblocked peptides were fitted with a red line. The open circles represent the theoretical values for linear (POG)_5_-(POG)_15_ crystal-derived models. The bottom panel compares the *R_g_* values against peptide length. The filled red circles represent the experimental *R_g_* values for the four collagen peptides (POG)_6-12_ and were fitted by a red line. (POG)_10unblocked_ is represented by a filled triangle. The open circles represent *R_g_* values calculated from linear crystal-derived models and were fitted by a black line.

Five standard collagen homotrimeric helices were selected for the present study. These comprised four blocked collagen peptides of increasing lengths: (POG)_6_, (POG)_8_, (POG)_10_, and (POG)_12_. In addition, one unblocked peptide, (POG)_10unblocked_, was studied in order to compare this with the blocked (POG)_10_ peptide to study the significance of end effects (Table 1). All five peptides formed a stable triple helical structure in solution [13,33]. Joint AUC sedimentation velocity (Figure 1) and SAXS (Figure 2) studies were performed to determine the hydrodynamic and X-ray scattering properties of these helices in solution. The sedimentation of an elongated macromolecule under high centrifugal force is dependent on its length and mass [34]. SAXS characterizes the average structure of an elongated molecule in solution in terms of its length, then the use of high resolution atomistic structures to model the low resolution scattering curves provides information on its flexibility [35].

**Table 1:** Summary of the AUC and SAXS data for (POG)_6_-(POG)_12_

Collagen peptide	Molecular mass**^1^** (Da)	*s*_(*20,w*)_ (S)		*R_g_* (nm)		*R* factor**^5^** (%)
		Experimental	Linear Model^2^	Experimental^3^	Linear Model^4^	
**(POG)_6_**	4989	0.83 ± 0.03	0.75	1.49 ± 0.02 1.67 ± 0.03	1.44 1.50	4.15
**(POG)_8_**	6594	0.95 ± 0.08	0.84	1.82 ± 0.03 1.89 ± 0.03	1.91 2.01	7.40
**(POG)_10_**	8196	1.07 ± 0.04	0.92	2.29 ± 0.02 2.38 ± 0.02	2.39 2.58	6.33
**(POG)_10unblocked_**	8070	1.19 ± 0.01	0.92	2.33 ± 0.05 2.45 ± 0.02	2.42 2.58	6.40
**(POG)_12_**	9801	1.16 ± 0.08	1.00	2.55 ± 0.04 2.61 ± 0.05	2.77 3.07	9.62

1 Molecular mass values were calculated from mass spectrometry data.

2 Predicted sedimentation coefficients for the linear crystal-derived models were calculated using HYDROPRO.

3 The first value was determined from Guinier *R_g_* anlysis; the second value was determined from GNOM *P*(*r*) analysis.

4 The first and second values were obtained from Guinier *R_g_* and GNOM *P*(*r*) analyses of the modelled *I*(*Q*) curves for linear (POG)_6_-(POG)_12_ crystal-derived structures. The same *Q* range was used for both experimental and modelled curves.

5 The *R* factors were calculated by comparing experimental *I*(*Q*) curves against modelled *I*(*Q*) curves for the linear crystal-derived structures.

### Sedimentation velocity analysis of collagen helices

Sedimentation velocity experiments were conducted on the five collagen model peptides. Absorbance sedimentation data were measured at three concentrations for each, and processed with SEDFIT software to give the size distribution functions *c*(*s*). The *c*(*s*) analyses resulted in good fits to the sedimentation boundaries and produced single distinct and narrow peaks for each collagen helix to show that these were structurally monodisperse with no dissociation as desired (Figure 1). No peaks were seen at the lowest S values that would suggest the presence of single dissociated helices. The experimental *s_20,w_* values were calculated from the peak positions in the *c*(*s*) analyses. The averaged *s_20,w_* values were 0.83 ± 0.03 S for (POG)_6_, 0.95 ± 0.08 S for (POG)_8_, 1.07 ± 0.04 S for (POG)_10_, 1.16 ± 0.08 S for (POG)_12_ and 1.19 ± 0.01 S for (POG)_10unblocked_ (Table 1). The averaged *s_20,w_* values increased with the number of (POG) repeats, which reflected the increasing mass and lengths of the helices. The peptide (POG)_10unblocked_ sedimented only slightly faster (0.12 S more) than the peptide (POG)_10_, suggesting that there were no significant difference in hydrodynamic properties between these two helices.

High resolution crystal structures were known for three standard collagen helices (POG)_n_, where *n* = 9, 10, and 11, and these revealed linear molecules [15,16]. These structures were used to derive starting structures that corresponded to each of the five peptides of the present study; these were designated as ‘crystal-derived structures’. Theoretical sedimentation coefficients $s_{20,w}^{0}$ were calculated from these crystal-derived structures using HYDROPRO. The AUC experimental data were compared with these calculated $s_{20,w}^{0}$ values to assess their divergence (Figure 2; Table 1). The experimental *s_20,w_* values were consistently larger for the five collagen helices compared with the theoretical values. The increasing divergence between the experimental and theoretical values of up to 0.27 S with peptide length suggested that the longer helices were less extended and linear than expected and corresponded to bent structures. Because the previously reported magnitude of the differences between HYDROPRO predictions and experimental values for macromolecules of well characterized *s_20,w_* values was typically ± 0.21 S [36], the low differences in the pairs of *s_20,w_* values for the (POG)_n_ peptides meant that additional experiments using SAXS were needed to confirm these solution structures.

### X-ray solution scattering of collagen helices

**Figure 3.**
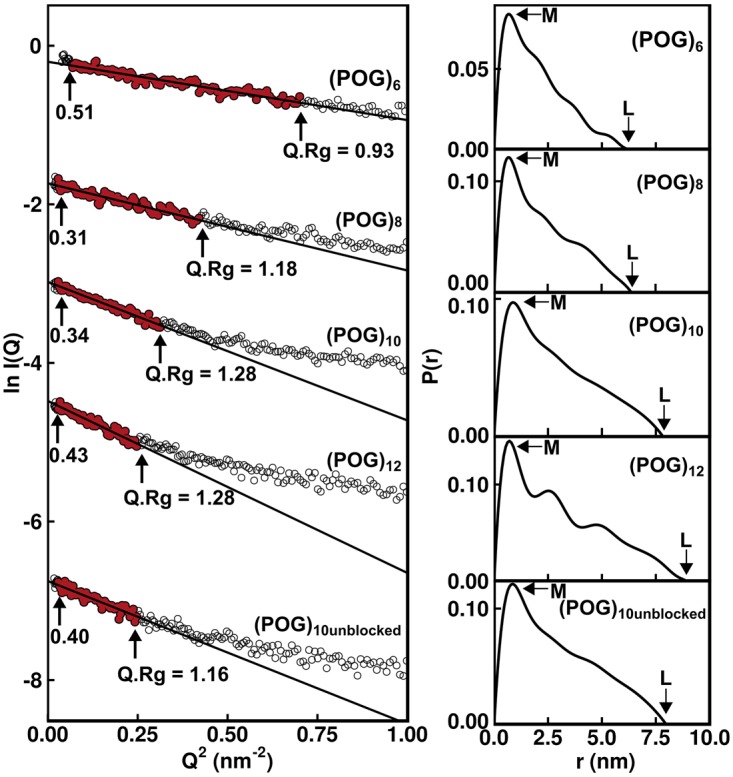
Experimental X-ray Guinier and *P*(*r*) analyses for the five collagen peptides. The Guinier radius of gyration *R_g_* plots at low *Q* values are shown on the left for five collagen peptides (POG)_6-12_ at concentrations of 0.8–1.0 mg/ml. The data points in red were fitted by best-fit lines which were used to calculate the *R_g_* values. For these, the *Q* ranges were 0.26–0.84 nm^−1^ for (POG)_6_, 0.17–0.65 nm^−1^ for (POG)_8_, 0.17–0.56 nm^−1^ for (POG)_10_, 0.17–0.50 nm^−1^ for (POG)_12_ and 0.17–0.50 nm^−1^ for (POG)_10 unblocked_. The *Q.R_g_* ranges are shown with each fit. The vertical axis is measured in arbitary units of ln *I*(*Q*). The right-hand panel displays the pair distribution analyses *P*(*r*) calculated from the experimental scattering curves. The *r* values of the maxiumum at *M* were 0.65 nm for (POG)_6_, 0.70 nm for (POG)_8_, 0.97 nm for (POG)_10_, 0.71 nm for (POG)_12_ and 0.88 nm for (POG)_10unblocked_. The maximum length *L* values were 6.5 nm for (POG)_6_, 6.4 nm for (POG)_8_, 7.8 nm for (POG)_10_, 8.9 nm for (POG)_12_ and 8.0 nm for (POG)_10unblocked_.

SAXS *I*(*Q*) data sets were obtained for the five collagen model helices. The Guinier analyses of these SAXS curves produced the radii of gyration *R_g_*, which was a measure of macromolecular extension (Figure 3). The Guinier regions became shorter with increased macromolecular extension. Therefore, the *Q* range of the Guinier fit was reduced successively from 0.26–0.84 nm^−1^ for (POG)_6_ to 0.17–0.50 nm^−1^ for (POG)_12_. The experimental *R_g_* values for the collagen helices were 1.49 ± 0.02 nm for (POG)_6_, 1.82 ± 0.03 nm for (POG)_8_, 2.29 ± 0.02 nm for (POG)_10_, 2.55 ± 0.04 nm for (POG)_12_ and 2.33 ± 0.05 nm for (POG)_10unblocked_ (Table 1). The increase in *R_g_* values with increasing peptide length agreed with the AUC results, which also showed an increase in *s_20,w_* values with collagen size. The similarity of the SAXS *R_g_* values for (POG)_10unblocked_ and (POG)_10_ agreed with the AUC data for these two peptides.

The experimental *R_g_* values were also calculated by an alternative approach based on pair-distance distribution analyses *P*(*r*) of the full *I*(*Q*) curves (Figure 3). This alternative set of *R_g_* values again increased with collagen size in a similar manner to the Guinier *R_g_* values (Table 1). The appearance of the *P*(*r*) curves was affected by noise in the *I*(*Q*) data sets that were attributed to the comparatively low molecular masses in use. These masses ranged from 4989 Da for (POG)_6_ to 9801 Da for (POG)_12_ when measured by mass spectrometry during their synthesis (Materials and Methods). Minor perturbations resulted in the form of small extra *P*(*r*) peaks for (POG)_12_ or an abrupt end to the *P*(*r*) curve for (POG)_10_ at large *r* values. Nonetheless, the *P*(*r*) analyses provided the maximum length *L* of each collagen helix which corresponded to the value of *r* when the *P*(*r*) curve reaches zero. The *L* values were 6.5 nm for (POG)_6_, 6.4 nm for (POG)_8_, 7.8 nm for (POG)_10_, 8.9 nm for (POG)_12_ and 8.0 nm for (POG)_10unblocked_. The increases in these *L* values were consistent with the increasing length of the collagen peptides. The *P*(*r*) peak maxima provided values for *M*, the most common interatomic distance in each collagen molecule, which reflects the width of the collagen triple helix. While the values of *M* shifted slightly, no correlation was seen with collagen length, as expected.

The experimental *R_g_* values were compared with the theoretical *R_g_* values calculated from the crystal-derived structures. The theoretical scattering curves were calculated from a coarse grained sphere model of our crystal-derived structures. The theoretical *R_g_* values were calculated from Guinier fits of the *I*(*Q*) curves in the same *Q* ranges that were used with the experimental curves for reason of consistency. The resulting *R_g_* values for the crystal-derived structures were 1.44 nm for (POG)_6_, 1.91 nm for (POG)_8_, 2.39 nm for (POG)_10_, 2.77 nm for (POG)_12_ and 2.42 nm for (POG)_10unblocked_ (Figure 2; Table 1). Comparison with the experimental *R_g_* values showed that the former were smaller than the theoretical *R_g_* values for the crystal-derived models, except for the smallest peptide (POG)_6_ (Figure 2). This increasing divergence with size indicated that, as the collagen peptides became longer, their solution structure became less extended. In addition, the theoretical *I*(*Q*) curves from the crystal-derived structures were compared against the experimental *I*(*Q*) curves. The goodness-of-fit *R* factors monitor the degree of deviation between the two curves. The observed *R* factors became higher with increased peptide length, these being 4.2% for (POG)_6_, 7.4% for (POG)_8_, 6.3% for (POG)_10_, 9.6% for (POG)_12_ and 6.4% for (POG)_10unblocked_ (Table 1). Given that the crystal-derived structures were very close to linear, the higher *R* factors and poorer curve fits with increasing peptide length suggested the presence of increased non-linearity in the longer helices. This result was consistent with the larger deviations seen between the experimental and theoretical *s_20,w_* values when the helices became longer (Table 1).

### Comparison of experimental data against MD ensembles

The full *I*(*Q*) curve out to large *Q* values contained further information on the solution structure of a macromolecule in addition to that on the overall structure provided by the *R_g_* values at low *Q* values. Comparison of the experimental SAXS curve with the theoretical SAXS curves calculated from stereochemically realistic atomistic conformations permitted the identification of the best fitting structural models. These conformations were determined from a 50 ns MD simulation at 300 K that produced 50 000 snapshots of stereochemically-correct structures for each of the collagen triple helices. These ensembles include a range of triple helical conformations, including non-linear molecules. In all five cases, the MD simulations resulted in an equilibrated system that contained the structures that were allowed by the force-field conformational space. As a check, simulations at 400 K did not change the distribution of the calculated *R_g_* values (see below). This indicated that the MD simulations reached equilibrium and had produced a suitably diverse pool of models. These models resulted from simulations using a physics-based all-atom explicit solvent force field, and thus sampled structurally representative conformations in solution. A theoretical *I*(*Q*) curve for every structural snapshot in the five MD simulations was calculated using coarse grained bead models of the atomic co-ordinates. Each of the resulting 50 000 curves was then compared against the corresponding experimental scattering curve. For this, the *R_g_* values were calculated for all 50 000 snapshots using the same *Q* range used for experimental Guinier fits (Figure 3), together with the 50 000 goodness-of-fit *R* factors between the experimental and theoretical *I*(*Q*) curves.

**Figure 4.**
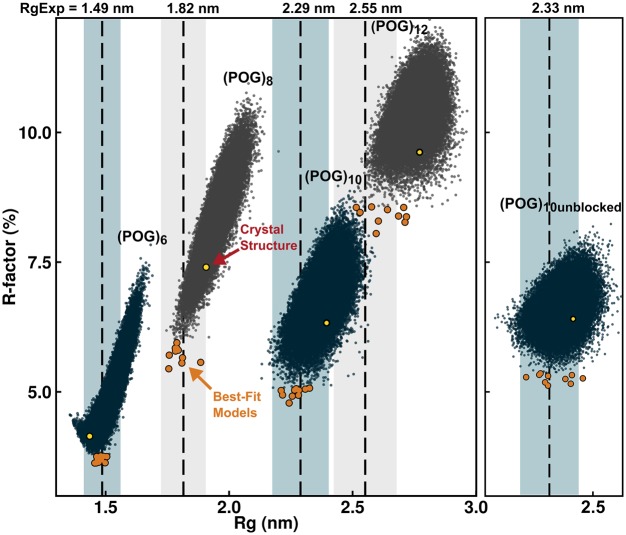
Comparison of the (POG)_6_-(POG)_12_ molecular dynamics ensembles with the X-ray scattering curves. The scattering curves *I*(*Q*) were calculated for each of the 50 000 structures created in the five molecular dynamics ensembles for (POG)_6_-(POG)_12_, following which the *R* factor and *R_g_* values for each structure were plotted. The dashed lines represent the experimental *R_g_* values for (POG)_6_-(POG)_12_, and the shaded bands represent a ±5% error range in the *R_g_* values. For each of the 50 000 structures, the ten best-fit structures with lowest *R* factors are shown in orange. The values for the four linear crystallographic models are shown in yellow.

**Figure 5.**
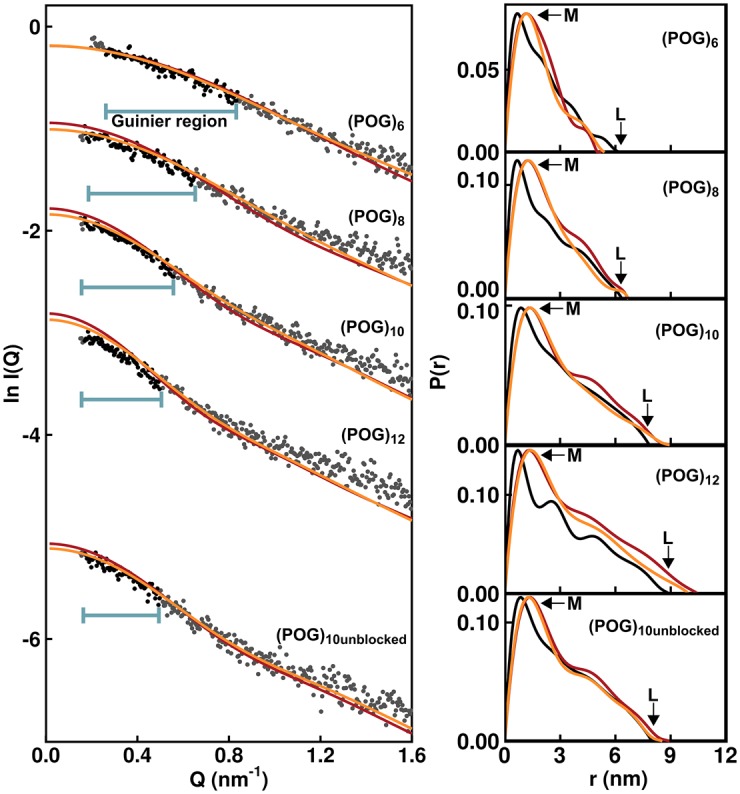
Overlap of the linear and best-fit modelled *I*(*Q*) curves for (POG)_6_-(POG)_12_ onto the X-ray scattering curves. The experimental *I*(*Q*) curves (black and grey circles) are compared with the MD best-fit (orange) and linear crystal-derived (red) modelled *I*(*Q*) curves for (POG)_6_-(POG)_12_. The horizontal bars indicate the region of each curve used to determine the *R_g_* values from Guinier analysis. The vertical axis is measured in arbitary units of ln *l*(*Q*). The right-hand panels show the overlaid pair distribution analyses of the experimental *I*(*Q*) curves (black), crystal-derived structure (red) and best-fit MD structure (orange).

For all five collagen peptides, many triple helix structures from the MD snapshots gave *R* factors that were lower than that seen for the crystal-derived structure (Figure 4). Ten optimal best-fit structures was identified for each peptide. The *R* factors for the 10 optimal structures (orange circles) were consistently lower than seen for the crystal derived triple helix models (yellow circles), indicating that these MD ensemble-derived structures gave a better fit to the experimental *I*(*Q*) data (Figure 4). The best-fit models (Figure 5, left, in orange) showed visually-improved *I*(*Q*) curve fits at the lowest *Q* values when compared with the crystal-derived linear structures (Figure 5, red). This improvement also reflected the reduced *R_g_* values of the longer POG peptides. This improvement in the curve fit based on the best-fit MD triple helix structures was not as clear for (POG)_12_ as for the three smaller triple helices, suggesting that a single structure for (POG)_12_ might not be a good representation of the solution average structure for this longest peptide. Views of the experimental and modelled *P*(*r*) curves (Figure 5, right) support the outcomes seen in the *I*(*Q*) fits. At large *r* values, where the *P*(*r*) curves contributed more strongly to the *R_g_* values, the orange best-fit curves were closer to the black curves than the red crystal-derived curves.

The *R* factors confirmed the above visual interpretation of the scattering fits. For the smallest triple helix (POG)_6_, the best fit MD structures resulted in *R* factors of 3.7% with *R_g_* values close to the experimental *R_g_*, indicating an excellent X-ray curve fit (Figure 5). The best fit triple helix structures were close to linear, which was consistent with the good agreement of the experimental (POG)_6_ data with the linear crystal derived scattering curves (*R* factor of 4.2%). For the three triple helices (POG)_8_, (POG)_10_, and unblocked (POG)_10_, the best-fit *R* factors were slightly higher at 4.8–5.4%. The best fit models showed *R_g_* values similar to the *R_g_* values seen experimentally, both of which were lower than expected from a linear crystal-derived structure (Table 1). The largest triple helix (POG)_12_ was the least well modelled by the simulations (Figure 4). The best-fit structures showed higher *R* factors of about 8.3% compared with the range of 3.6–5.4% for the lowest *R* factors for the four shorter peptides, suggesting that this peptide showed the most flexibility. Nonetheless, the best-fit structures for (POG)_12_ possessed *R_g_* values close to the experimental *R_g_* value, and, again, these values were lower than observed for the linear crystal-derived structure.

**Figure 6.**
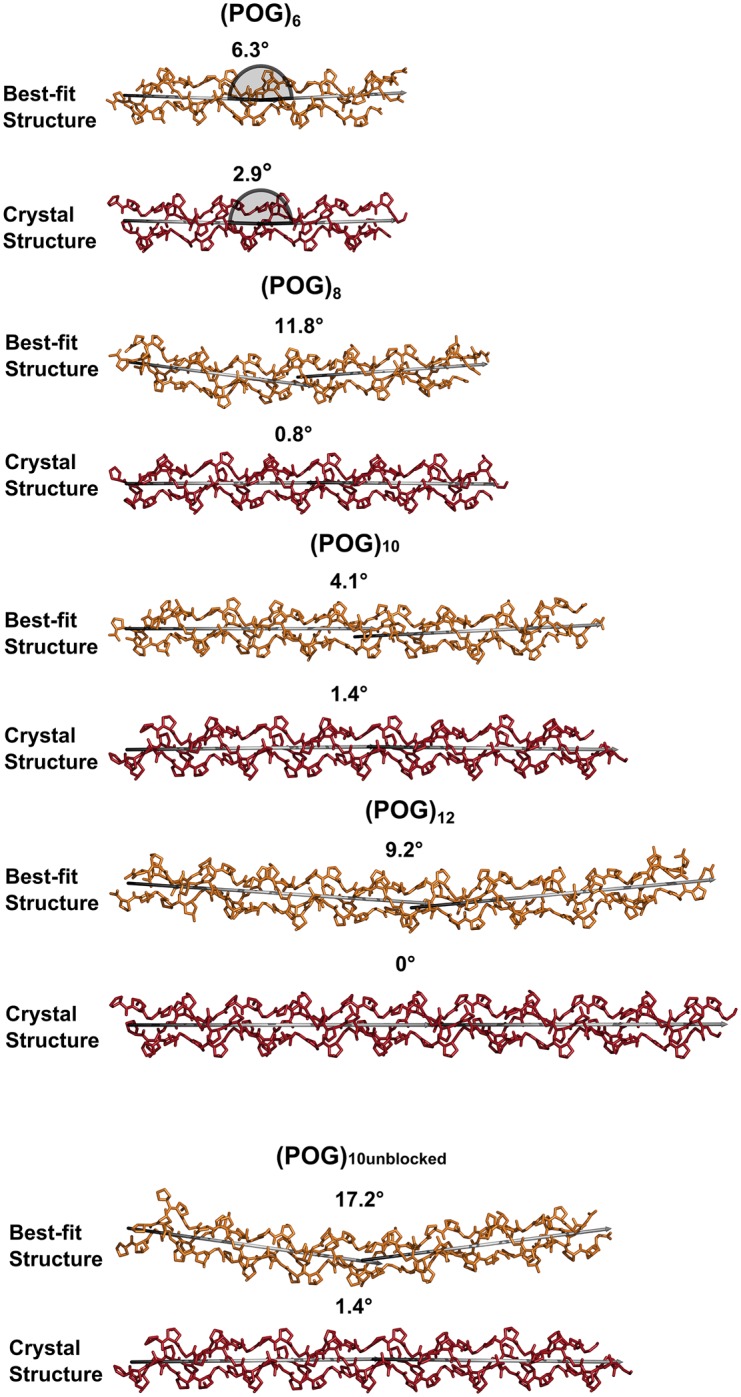
Comparisons between the scattering best-fit and linear crystal-derived models. The three polypeptide chains of the best-fit structures for (POG)_6_-(POG)_12_ are shown in orange, while those for the linear models are shown in red. In order to monitor the degree of bend in these structures, two verticies shown in grey were fitted to the N-terminal and C-terminal halves of the collagen triple helix, and the angle between the two verticies is displayed above each structure, with 0° representing a linear structure. The grey half circles for (POG)_6_ define how the angles were measured.

**Figure 7.**
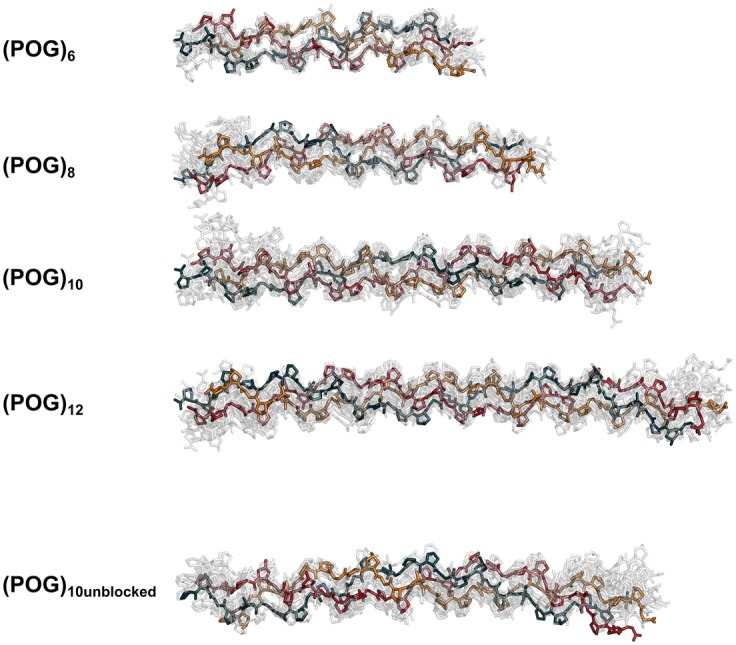
Superimposition of the ten best-fit structures for (POG)_6_-(POG)_12_. Stick representations of the ten best-fit models from each scattering fit analysis were superimposed upon each other. The best-fit structure in each is opaque, with each polypeptide chain represented in blue, red and orange. The remaining nine structures were rendered semi-transparent. Hydrogen atoms were not shown for reason of clarity.

Visual examination and analysis of the best fit triple helix structures for each collagen peptide were carried out. As expected from the lower *R_g_* values, the longer peptides showed modest non-linearity. The comparison of the single best fit MD ensemble structure with the crystal-derived structure indicated an increased amount of non-linearity that appeared as a subtle bending of the triple helix (Figure 6). To visualise the degree of conformational variation, the single best-fit MD structure (bold color) was superimposed upon the best ten MD structures (gray) (Figure 7). The smallest peptide (POG)_6_ showed the least diversity, with all ten structures clustering around the best-fit structure. The other peptides showed somewhat more conformational variability, with the largest deviations seen at the N- and C- termini.

## Discussion

In order to understand collagen flexibility better, we have here reported a multidisciplinary experimental study on varying lengths of collagen triple helices composed of the most stable Pro-Hyp-Gly units, the results of which were compared with atomistic MD simulations. First, complementary AUC and SAXS data indicated that the shortest (POG)_6_ triple helix was linear, while the longer triple helices were increasingly less well explained by a linear structure. Second, the MD simulations identified best-fit atomistic models from a large ensemble of physically-realistic triple helices at computational equilibrium. The bent best fit MD models showed notably better agreement with the experimental data than that from linear models derived from crystal structures. The decrease in the *R_g_* values of these best fit models compared with the *R_g_* value of the linear model indicated bending or flexibility that had not been previously characterised at the molecular level. While all the *R* factors between the best fit MD models and experimental scattering curves were reasonable, the final best-fit *R* factors increased with longer triple helix length. This outcome indicated that, not only did the crystal-derived linear structures became even less appropriate to describe the collagen scattering curves with increase in length, but also that a single ensemble of related MD structures did not represent as well the average solution structure for the longer triple helices as their lengths increased; this also indicated flexibility. The extrapolation of our best-fit (POG)_12_ models to create longer theoretical triple helices indicate that bending and flexibility would be present in a molecule similar in length to type I collagen. It is important to note that type I collagen, (Xaa-Yaa-Gly)_338_, has a much lower imino acid content than (POG)_n_, with Pro in the Xaa position of only 1/3 of the collagen triplets; Hyp in the Yaa position of 1/3 of the triplets; and 10% of all triplets being Pro-Hyp-Gly. In addition to the bending reported here, real collagen sequences with a decreased proportion of imino acids will introduce additional conformational flexibility through twist variability, a feature reported in crystal structures of collagen peptides [37]. It is likely that variability in twist will also be linked with bending. Further studies would be needed on real collagen sequences to evaluate the impact of local collagen regions with low imino acid sequences on the flexibility of the collagen triple helix. In addition to variability in these triple helix features, it is likely that flexibility will also occur in non-fibrillar collagens at the numerous sites where the repeating (Xaa-Yaa-Gly)_n_ sequence is interrupted.

Traditionally the collagen molecule is regarded as a prototype of a rod-like protein with little flexibility. In collagen, the φ and ψ angular range of Pro and Hyp residues is extremely limited, in contrast with the large area of the Ramachandran plot available to Gly residues. At each axial level in the three staggered chains in the triple helix, a Gly residue in one chain is positioned at the same level as a Xaa residue from the second chain and a Yaa residue from the third chain. Features thought to confer rigidity to the triple helix include the high imino acid content and the tight supercoiling of the three chains mediated by interchain hydrogen bonding, which makes the triple helix resistant to digestion by most enzymes. In spite of these rigidity factors, data sets from experimental and computational studies have indicated some overall flexibility of the triple helix as well as instances of site-specific bending. For example, rotary shadowing electron microscopy showed a kink corresponding to the imino acid-poor collagenase cleavage site for type I collagen; many flexible sites were seen in type IV collagen, which may correspond to interruptions in the Xaa-Yaa-Gly repeat important for the basement membrane network structure [9]. In addition, a pronounced kink was visualized within the heterotrimeric triple helix of complement protein C1q that creates the bouquet-like structure considered essential for efficient immune interactions [38]. It is important for collagen function to define the sites of non-linearity and flexibility in molecular terms within the triple helix, and establish their relationship to enzyme susceptibility, higher order structure, and other biological features. The MD structures from the present study (Figure 7) now offer a means of characterizing these sites in molecular terms, and testing these against experimental data to validate them.

The experimental results showed that, even for the most imino acid-rich collagen sequence that forms the most stable triple helix as represented by the POG repeats, a small degree of non-linearity was observed in solution for the longer peptides. Visual inspection of the best fit MD models indicated the presence of some curvature in the triple helix. In order to quantify this bending, the N-terminal and C-terminal halves were taken to be linear segments, and the angle between these two halves was measured (Figure 6). This approximate bending angle in the best fit MD structures varied between 4° and 17° for the five peptides (with 0° being a perfect linear model). This contrasts with the relatively linear conformations seen for the crystal-derived structures (0°–3°) using the same method (Figure 6). Therefore, our data supported a bending in solution greater than that seen in the crystal structures for these (POG)_n_ peptides. As a control, our results for (POG)_10unblocked_ were compared with the blocked (POG)_10_ to clarify potential end effects. There was little difference between the blocked and unblocked helices in the AUC or SAXS data, suggesting similar hydrodynamic properties. However, the terminal regions showed the largest variation when the top ten best fit structures for all peptides longer than (POG)_6_ were superimposed (Figure 7), consistent with the terminal disorder seen in crystal structures and NMR studies [39].

Interestingly, a small amount of molecular bending similar to that observed for our (POG)_n_ helices in solution, has been reported in four crystal structures of triple helical peptides containing sequences other than Pro-Hyp-Gly, either alone or in complex with another protein. For instance, the integrin binding peptide (GPO)_3_GFOGER(GPO)_3_ alone showed ‘junctional kinking’, with an ∼8° angle between the N-terminal (GPO)_3_ and the central GFOGER sequence, and a ∼9° angle between the C-terminal (GPO)_3_ and the central GFOGER [24]. These angles were increased considerably to ∼16° and ∼14.5° when the same peptide was complexed with the integrin I domain [40]. Bends of similar magnitude within the triple helix were reported in the high resolution crystal structures for an imino acid poor peptide [12] (POG)_3_ITGARGLAG(POG)_4_, a triple helical peptide complexed with MMP (GPO)_3_GPQGLAGQRGIVGLOGQRGER(GPO)_3_ [41], and a peptide complexed with the *Streptococcus aureus* collagen binding protein CNA, (GPO)_4_GPRGRT(GPO)_4_ [42]. The bending seen in these triple helices is most likely to be related to their amino acid sequence. In comparison with our present study, our results confirm that bending occurs in (POG)_n_ helices and not just to non-POG helices. It will also be important to determine how Xaa-Yaa-Gly sequences, which are less stable than POG sequences, will affect the flexibility and specific binding sites in triple helices.

The biological significance of non-linearity in the collagen triple helix in both standard POG and non-POG sequences will be manifested in the context of longer collagen molecules. The collagen triple helix characterized here is formed from only Pro-Hyp-Gly units and is likely to be the most rigid triple helix formed with natural amino acids. Non-linearity within the collagen molecule can affect its biosynthesis and secretion, its interactions with other matrix proteins and enzymes, and the formation of complete collagen fibril structures and tissue mechanics. Even a low level of bending in a full-length collagen helix could provide sufficient flexibility to allow collagen molecules to be transported through the Golgi or could create fibrils that can withstand forces that may break a more brittle structure. As an example, the low angle X-ray diffraction pattern from rat tail tendon indicated that the collagen molecules cannot be straight within the fibril, and may kink as they pass through the less dense gap region [43]. In addition, all non-fibrillar collagen types and collagen domains in proteins such as C1q and mannose binding lectin contain breaks in the repeating (Xaa-Yaa-Gly)_n_ sequence, which are likely to lead to kinks and flexible sites [9,44]. Flexibility in the C1q and mannose binding lectin collagen helices are likely to facilitate immune function through their binding to flexible multi-domain ligands, as recently exemplified by the MASP solution structure [45]. This will contribute to their function in activating the complement pathways of immune defense. Kinks have also been reported for several collagens with mutations leading to the bone disorder osteogenesis imperfecta [46–48]. These disease-associated mutations are likely to perturb the regular packing of the collagen helices in bone through excessive bending. The present work opens the way for further molecular studies to investigate collagen flexibility at biologically important sites and mutation sites.

Our multidisciplinary methods provide a new approach to investigating the flexibility of the collagen triple helix in solution, namely the combination of AUC and SAXS with MD simulations. This joint approach has been applied to other proteins during the past decade [18,19,45] and is indeed being adopted by other groups [49]. Both AUC and SAXS provided shape parameters in the *s_20,w_* and *R_g_* values respectively that monitored the overall length of the helices. AUC demonstrated through the single *c*(*s*) peaks that only triple helices had formed in solution, while SAXS provided the diffraction data for modelling fits. The AUC and SAXS data were fitted using the generation of physically-realistic all-atom structures from MD starting from known crystal structures [19]. The collagen helices were modelled using all-atom explicit solvent MD simulations using a physics-based force field. Our MD simulations were performed starting from a standard triple helix geometry; at MD equilibrium, these resulted in 50 000 structures for each of the five helices. Following calculation of the SAXS curves [31], each set of structures was compared with experiment to confirm the bending of the collagen helices at a molecular level, quantify the extent of bending, and provide molecular structures for these (Supplementary Information). Utilizing varying lengths of helices enabled the conformation of the helices to be examined as their lengths increased. In conclusion, our joint AUC-SAXS-MD approach complements previous methods used to study collagen flexibility, and is carried out in solution under physiological conditions. Because physically realistic full structures were used, this approach has high potential to answer further questions relating to the collagen triple helix sequence, stability and flexibility.

## Abbreviations

AUC: analytical ultracentrifugation
MD: molecular dynamics
POG: the tripeptide sequence Pro-Hyp-Gly
SAXS: small angle X-ray scattering
